# Genome‐Wide Association Analyses Reveal the Genetic Basis of EMS Mutagenesis Efficiency in Rice

**DOI:** 10.1002/advs.202517647

**Published:** 2025-11-20

**Authors:** Peizhou Xu, Qinglu Zhang, Le Xue, Zhen Zhang, Nanhui Luo, Zhaoyang Cheng, Duo Xia, Shuqin Zheng, Xianjun Wu, Hao Zhou

**Affiliations:** ^1^ State Key Laboratory of Crop Gene Exploration and Utilization in Southwest China Rice Research Institute Sichuan Agricultural University Chengdu 611130 China; ^2^ National Key Laboratory of Crop Genetic Improvement Hubei Hongshan Laboratory Huazhong Agricultural University Wuhan 430070 China

**Keywords:** antioxidant capacity, EMS mutagenesis, mutation breeding, oryza sativa, Rc gene

## Abstract

Ethyl methanesulfonate (EMS) mutagenesis is widely used to generate genetic diversity in crops, but its efficiency is strongly genotype‐dependent, and which underlying mechanisms remain poorly understood. Here, a large‐scale phenotypic analysis of 420 diverse accessions of rice (*Oryza sativa* L.) is performed, revealing extensive variation in EMS tolerance (survival rate) and mutagenesis efficiency (mutation frequency). The *Aus* subpopulation consistently outperformed others for both traits. A genome‐wide association study (GWAS) identified a major locus, *SRD7*, linked to reduced survival rate under EMS stress. Within this locus, the *Rc* gene, a key regulator of proanthocyanidins biosynthesis, is identified as the candidate causal factor. Haplotype analysis showed that functional *Rc* alleles confer high EMS tolerance, a conclusion further validated using transgenic knockout lines. Using near‐isogenic lines (NILs), it is confirmed that *Rc* not only improves seed survival after EMS treatment, but also unexpectedly increases genome‐wide mutation frequency. Mechanistic studies demonstrated that *Rc* enhances the antioxidant capacity of seeds by elevating CAT, SOD, and POD activities, while reducing H_2_O_2_ accumulation, thereby alleviating EMS‐induced oxidative damage. The findings establish *Rc* as a pleiotropic regulator that enhances EMS mutagenesis efficiency, providing a feasible strategy for accelerating the development of improved rice germplasm.

## Graphical abstract

Based on large‐scale screening of 420 rice accessions, GWAS identified the *Rc* locus as a key regulator of EMS mutagenesis efficiency. Functional Rc alleles enhance both seed survival and genome‐wide mutation frequency by boosting antioxidant enzyme activities (CAT, SOD, POD) and reducing oxidative damage. This pleiotropic role provides a strategic approach for optimizing mutagenesis and accelerating rice germplasm improvement.

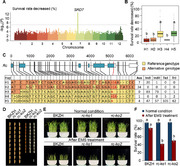

## 1 Introduction

Global food security faces unprecedented challenges from climate change, resource scarcity, and population growth because the world's population is projected to reach 9.7 billion by 2050.^[^ ^1^ ^]^ To meet this demand, crop breeding must use highly diverse germplasm to develop more sustainable, resilient, and high‐yielding varieties. Mutation breeding, as an established approach that accelerates the natural mutation process, has played a critical role in generating genetic diversity for crop improvement. More than 3,400 officially released mutant varieties across 242 species, including staple crops with enhanced yield, stress tolerance, and disease resistance, highlight the global impact of mutation strategy (FAO/IAEA Mutant Variety Database; MVD). Landmark mutant examples of semi‐dwarf rice ‘Calrose 76’ and barley ‘Diamant’ have revolutionized agriculture by improving lodging resistance to largely elevate crop yield and quality.^[^ ^2^, ^3^ ^]^ Among physical and chemical mutagens, *ethyl methanesulfonate (EMS)* is especially valuable for its operational simplicity, high mutation density, and broad utility, which has accounted for >80% of chemically induced mutants in MVD, so that it is ideal for studying functional genomics and breeding efficiency by creating broad allelic series.^[^ ^4^, ^5^ ^]^

Rice (*Oryza sativa* L.), a model grass crop with a well‐annotated genome, exemplifies the dual value of EMS mutagenesis in both basic research and applied breeding. Its compact genome facilitates gene discovery, while its role as a global staple crop drives strong demand for improved varieties with desirable traits. EMS‐induced mutant populations have supported two major research directions: 1) the creation of elite traits—for example, rapid generation of agriculturally valuable mutant phenotypes such as salt‐tolerant mutants;^[^ ^6^ ^]^ and 2) the acceleration of functional genomics studies through reverse genetics platforms (e.g., TILLING) and high‐throughput sequencing‐based BSA methods (such as MutMap).^[^ ^7^, ^8^, ^9^ ^]^ However, a critical bottleneck remains: the efficiency of EMS mutagenesis is highly genotype‐dependent. Mutation frequency, plant survivability, and physiological damage vary widely among rice varieties after EMS treatment, and the underlying mechanisms remain poorly understood, limiting their broad application across diverse genetic backgrounds.^[^ ^10^, ^11^ ^]^

Although EMS mutagenesis follows a relatively conserved molecular mechanism—primarily the alkylation of guanine leading to G:C > A:T base transitions, it also exhibits strong species‐ and genotype‐specific responses.^[^ ^12^, ^13^ ^]^ In Arabidopsis and barley, for instance, genetic background significantly influences mutation density and plant tolerance, where diverse accessions differ in survival rates.^[^ ^14^, ^15^ ^]^ In rice, however, a systematic large‐scale assessment of EMS responses across diverse germplasms remains lacking. It is unclear whether mutation frequency and physiological tolerance are governed by shared regulatory mechanisms or controlled independently.

In a preliminary study, we have observed striking differences in survival rate (SR) and mutation frequency (MF) between the *indica* variety Yuehuangjinzhan (YHJZ) and the *japonica* variety K9553 after EMS treatment, suggesting genetic control of these traits. Based on this observation, we systematically analyzed EMS responses in a panel including 420 genetically diverse rice accessions collected worldwide, and quantified SR, MF, and physiological damage. Genome‐wide association studies (GWAS) and haplotype analysis identified key loci regulating SR and MF. Among the loci, *Rc*, a gene classically associated with pericarp pigmentation, was revealed to pleiotropically regulate both EMS tolerance and mutation frequency by enhancing antioxidant capacity. Based on the findings, we propose a practical “high‐efficiency mutagenesis” screening strategy to identify optimal parental genotypes for breeding. By establishing a genetic link between natural variation and EMS mutagenesis efficiency, this study provides both mechanistic insights and a practical framework for precision mutation breeding to accelerate genetic improvement in rice.

## 2 Results

### 2.1 The Efficiency of EMS Mutagenesis Varies among Rice Accessions

**Figure 1:**
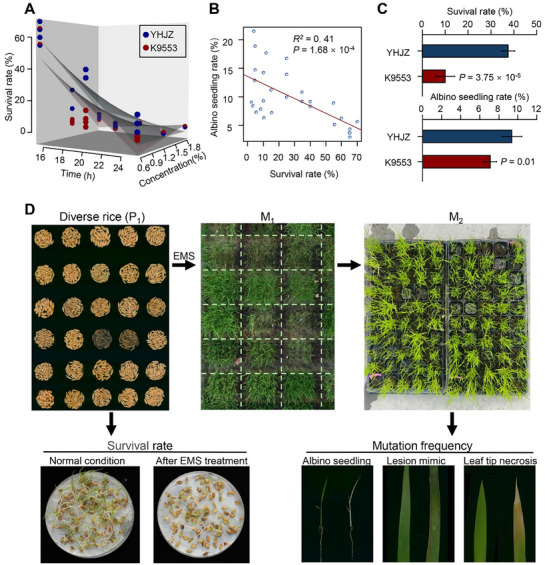
Phenotypic characterization of EMS mutagenesis tolerance and mutation frequency in diverse rice germplasm resources. A) 3D plot illustrating the regression relationships among seed survival rate, treatment duration, and EMS concentration. Blue and red points represent the phenotypic values of YHJZ and K9553, respectively, while the corresponding regression surfaces are shown in matching colors. B) Regression relationship between M2 mutation frequency and M1 seed survival rate under different EMS treatment conditions. C) Comparison of M1 seed survival rate and M2 mutant frequency between YHJZ and K9553 following treatment with 0.6% EMS for 20 h. *P‐*values were calculated using two‐tailed Student's *t*‐tests. D) Schematic workflow illustrating the evaluation of EMS mutagenesis efficiency across diverse rice germplasm resources.

To assess EMS mutagenesis efficiency, we treated two rice varieties using EMS solutions with different concentrations and durations (Table S1, Supporting Information). Survival rate of M₁ plants sharply decreased with an increase in EMS concentration and treatment time (**Figure** **1**A). In contrast, the frequency of albino seedlings in the M_2_ generation was negatively correlated with M₁ survival rate of M₁ plants (*r^2^* = 0.41, *P* = 1.68 × 10^−4^) (Figure 1B). Across nearly all treatment conditions, the *indica* variety YHJZ exhibited significantly higher M₁ survival rates and higher M_2_ albino seedling frequency than the *japonica* variety K9553, indicating greater EMS mutagenesis efficiency in YHJZ (Figure 1C).

**Figure 2:**
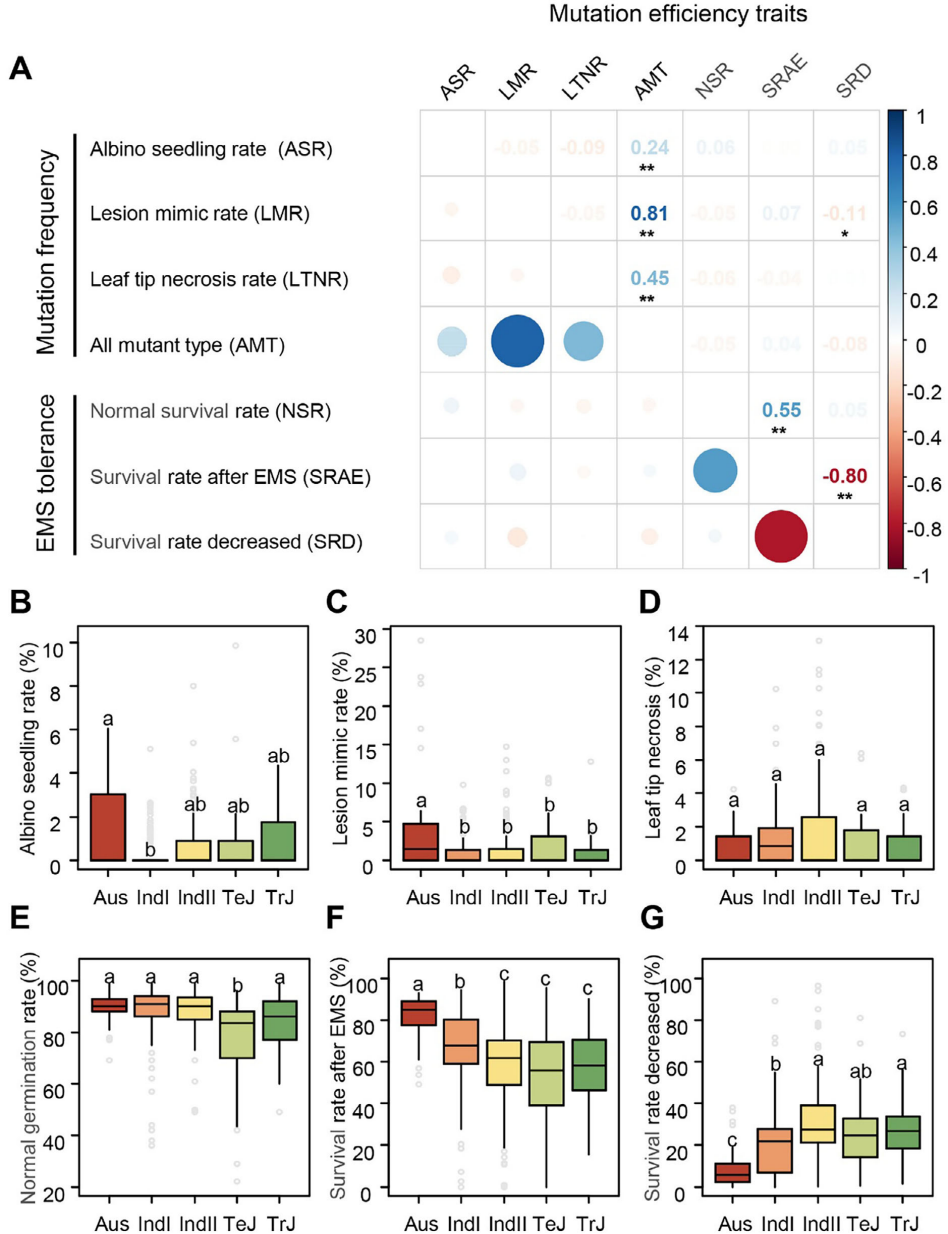
Phenotypic variation and correlation of EMS mutagenesis‐related traits in rice. A) Correlation matrix of EMS mutagenesis efficiency traits across diverse rice germplasm. * and ** denote significance at *P* < 0.05 and *P* < 0.01, respectively. B–G) Phenotypic distribution of EMS‐induced mutation frequency (B‐D) and EMS tolerance traits E–G) across different subpopulations of cultivated rice. Distinct lowercase letters indicate significant differences at the 5% level (*P* < 0.05) according to Duncan's multiple range test.

To systematically dissect the genetic basis of EMS mutagenesis efficiency, we analyzed a diverse rice germplasm panel. Two key traits were quantified: 1) EMS tolerance, measured as the reduction of survival rates before and after EMS treatment, where smaller reductions indicate higher tolerance (Figure 1D), and 2) mutation frequency, measured as the proportion of M_2_ seedlings displaying mutant phenotypes, such as albinism, lesion mimic, or leaf tip necrosis. High EMS tolerance of a genotype combined with a high proportion of phenotypic mutants in M_2_ generation reflects high mutagenesis efficiency.

For large‐scale analysis, we employed the treatment condition with 0.6% EMS for 20 h, which maximized phenotypic differences between *indica* YHJZ and *japonica* K9553 previously. Detailed phenotypic data are presented in Table S2 (Supporting Information). Among 420 diverse rice accessions, tolerance‐related traits (normal and post‐mutagenesis survival rates) showed positively skewed distributions, whereas mutation frequency‐related traits (albino rate, lesion mimic rate, and leaf tip withering rate) displayed negatively skewed distributions (Figure S1, Supporting Information). No significant correlation was detected between tolerance traits and mutation frequency traits, nor among different mutation classes. The only significant association was a positive correlation between normal survival rate and post‐mutagenesis survival rate (**Figure** **2**A).

Consistent with previous studies, the *indica* variety YHJZ exhibited both higher tolerance and higher mutagenesis frequency than the *japonica* variety K9553, suggesting a species differentiation in EMS mutagenesis efficiency. To test this, we compared subpopulations of the cultivated rice. Among mutation frequency‐related traits, the *Aus* subpopulation showed significantly higher albino and lesion mimic rates than other subpopulations of *IndI*, *IndII*, *TeJ*, and *TrJ* (Figure 2B–D). For tolerance‐related traits, *Aus* accessions significantly had not only higher post‐mutagenesis survival rates, but also lower reductions of survival rate than other groups (Figure 2E–G). Collectively, these results identify the *Aus* subpopulation as having the most efficient EMS mutagenesis performance among all rice groups examined.

### 2.2 Mutant Sequencing Reveals Higher Mutagenesis Frequency in *Aus*

**Figure 3:**
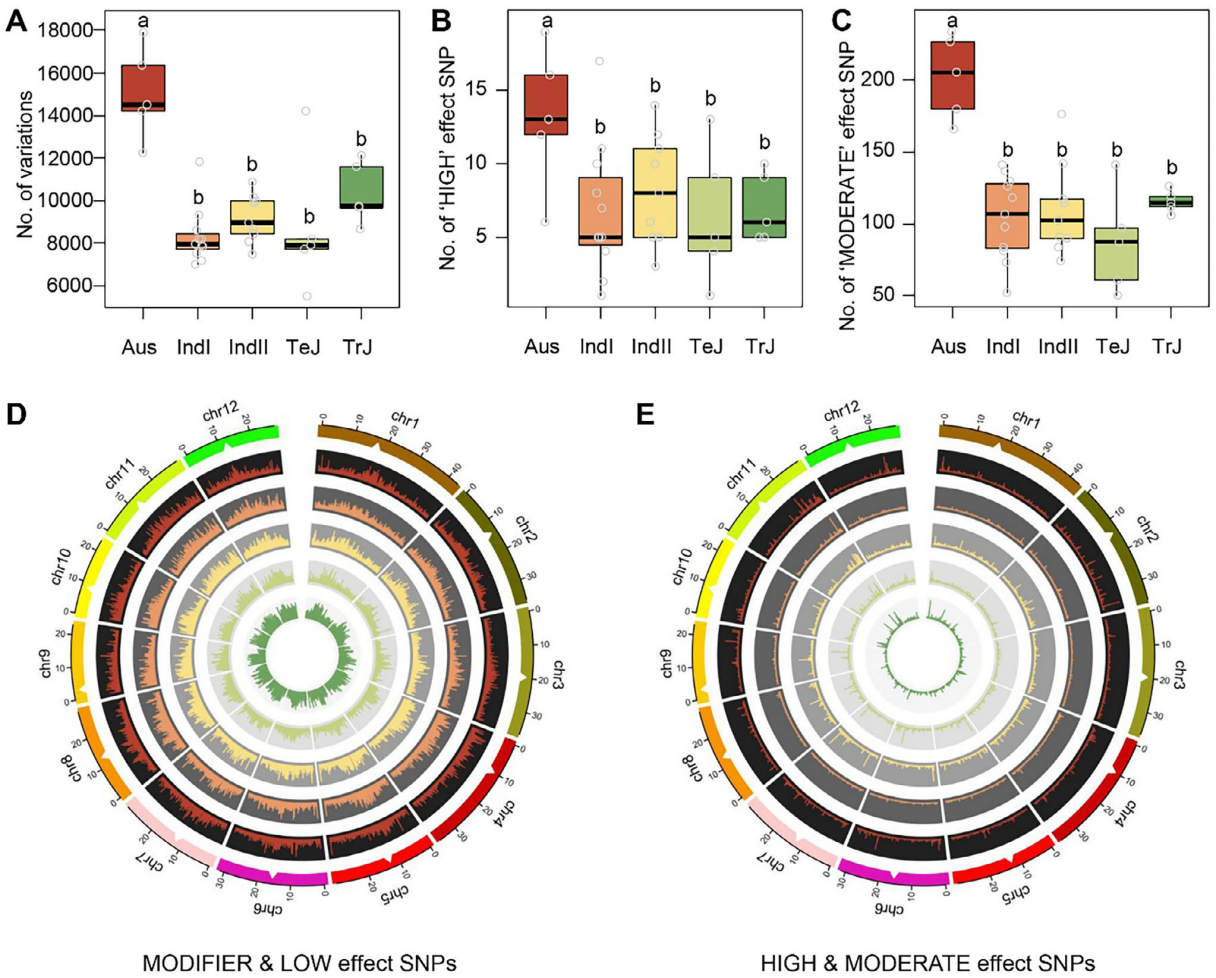
Genome‐wide distribution of EMS‐induced mutation sites in rice. A–C) Comparison of total EMS‐induced SNPs (A), 'HIGH' impact SNPs (B), and 'MODERATE' impact SNPs (C) among M_1_ populations from different subpopulations after EMS mutagenesis. Lowercase letters indicate significant differences at the 5% level (*P* < 0.05) according to Duncan's multiple range test. D,E) Chromosomal distribution of EMS‐induced 'MODIFIER & LOW' impact SNPs (D) and 'HIGH & MODERATE' impact SNPs (E) among subpopulations. From the outermost circle inward, the tracks represent chromosomes, and five subpopulations (Aus, IndI, IndII, TeJ, and TrJ, respectively). The height of each line in each circle indicates SNP frequency within genomic regions for each subpopulation. Definitions of ‘HIGH’, ‘MODERATE’, ‘LOW’, and ‘MODIFIER’ effect SNPs are provided in the Experimental Section.

To investigate the effects of genetic backgrounds on EMS mutagenesis, we performed whole‐genome sequencing of M_1_ seedlings (pooled five plants per accession) from five rice subpopulations. The *Aus* subpopulation displayed a significantly higher number of EMS‐induced genome‐wide SNPs than the others of *IndI*, *IndII*, *TeJ*, and *TrJ*) (**Figure** **3**A). Functional annotation of SNPs using snpEff showed that *Aus* accessions accumulated significantly more SNPs classified as ′HIGH′ and ′MODERATE′ impact variants than the others, consistent with the elevated mutation frequencies observed in their M_2_ generation (Figure 3B, C).

We further examined chromosomal distributions of EMS‐induced variants. Weak‐impact variants (MODIFIER and LOW) were enriched near centromeres, whereas strong‐impact variants (HIGH and MODERATE) were more abundant in distal chromosomal regions (Figure 3D, E). Overall variant density was also higher near centromeres, likely reflecting higher homologous repair efficiency in euchromatic regions versus heterochromatic regions, which is consistent with previous studies.^[^ ^16^ ^]^ Notably, high‐impact variations formed distinct chromosomal hotspots that partially overlapped with gene‐dense regions (Figure S2, Supporting Information), consistent with recent findings in rice.^[^ ^12^ ^]^

### 2.3 Natural Variation of Rc Influences EMS Tolerance in Rice

To uncover genetic determinants of EMS mutagenesis efficiency in cultivated rice, we conducted a genome‐wide association study (GWAS) on tolerance‐ and mutation frequency‐related traits across 420 diverse rice accessions. Because most phenotypic traits deviated from normality, the BOX‐COX transformation was applied prior to GWAS for reducing false positives.^[^ ^17^ ^]^ No significant loci were identified for mutation frequency traits, suggesting that phenotypes such as albinism, lesion mimicry, and leaf‐tip necrosis in M_2_ plants are not controlled by a small set of major loci (Figure S3 and Table S3, Supporting Information). In contrast, multiple loci significantly associated with tolerance traits were identified. Specifically, eight significant loci for normal survival rate were mapped to chromosomes 1, 2, 3, 4, 6, 7, 9, and 11, and only one locus each was identified for post‐EMS survival rate on chromosome 5 and survival rate reduction ratio on chromosome 7.

**Figure 4:**
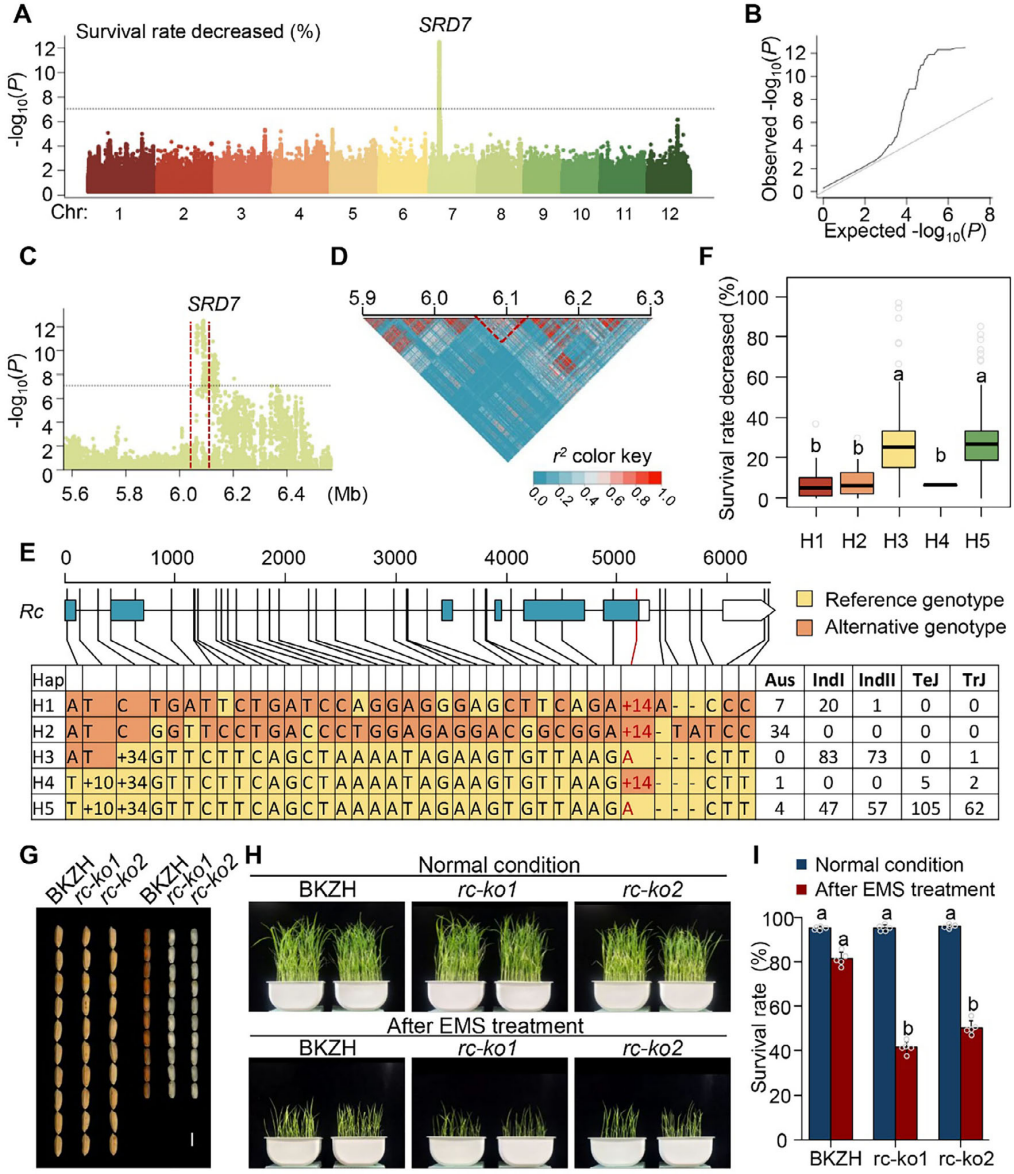
Natural variation in the *Rc* gene influences rice seed tolerance to EMS. A,B) Manhattan (A) and quantile‐quantile (QQ) (B) Plots showing genome‐wide association results for the reduction in survival rate after EMS treatment using a mixed linear model. C) Regional Manhattan plot of the 400‐kb region surrounding the associated locus *SRD7*. D) Linkage disequilibrium (LD) heatmap of the same 400‐kb genomic region. E) Haplotype analysis of the candidate gene *Rc*, highlighting a functional 14‐bp insertion/deletion in exon 6. F) Comparison of the reduction in survival rate among accessions carrying different *Rc* haplotypes. G) Comparison of seed and brown rice appearance between BKZH and *Rc*‐knockout lines (*rc‐ko1* and *rc‐ko2*), where scale bar = 5 mm. H) Representative seedling survival images before and after EMS mutagenesis. I) Comparison of seedling survival rates under normal conditions and after EMS mutagenesis. Distinct lowercase letters in (F) and (I) indicate significant differences at the 5% level (*P* < 0.05) according to Duncan's multiple range test.

Among all the associated loci, the most significant locus, designated *SRD7*, was located on chromosome 7 and associated with the survival rate reduction ratio (**Figure** **4**A–C). Linkage disequilibrium (LD) analysis refined the candidate region to a 6.06–6.12 Mb block containing eight genes (Figure 4D). Within this region, among the eight genes, *LOC_Os07g11020* (*Rc*), previously known to control pericarp pigmentation and seed dormancy in rice,^[^ ^18^ ^]^ emerged as a strong candidate. Since seed dormancy is correlated with survival rate and *Rc* promotes the accumulation of proanthocyanidins (compounds with antioxidant activity that may reduce EMS‐induced oxidative damage), we conducted haplotype analysis on *LOC_Os07g11020* (Figure 4E). Based on 20 SNPs and two InDels, five major haplotypes were classified (H1–H5). Previous studies^[^ ^19^ ^]^ show that a 14‐bp deletion in exon 6 abolishes *Rc* function, rendering haplotype H3 and H5 non‐functional, whereas H1, H2, and H4 are functional. We observed a subpopulation‐specific distribution of the haplotypes; for example, H2 occurred exclusively in the Aus group.

Phenotypic comparisons revealed that functional haplotypes H1, H2, and H4 conferred significantly smaller reductions in survival rate after EMS treatment than non‐functional haplotypes H3 and H5 (Figure 4F). These results indicate that natural variation at *Rc* may contribute to the differences in EMS tolerance among rice accessions.

### 2.4 Rc Enhances EMS Mutagenesis Efficiency by Improving Antioxidant Capacity in Rice Seeds

To genetically validate the role of *Rc* in EMS tolerance, we generated *Rc* knockout lines in the red‐pericarp *indica* variety BKZH using CRISPR/Cas9 (Figure S4, Supporting Information). Two independent knockout lines (*rc‐ko1* and *rc‐ko2*) were obtained, both exhibiting a clear white pericarp phenotype in contrast to the red pericarp of wild‐type BKZH (Figure 4G). Under control conditions, seedling survival rates were similar among the genotypes. However, following EMS treatment, both knockout lines exhibited a significant reduction in seedling survival compared to BKZH, confirming that *Rc* plays a critical role in enhancing EMS tolerance (Figure 4H, I).

**Figure 5:**
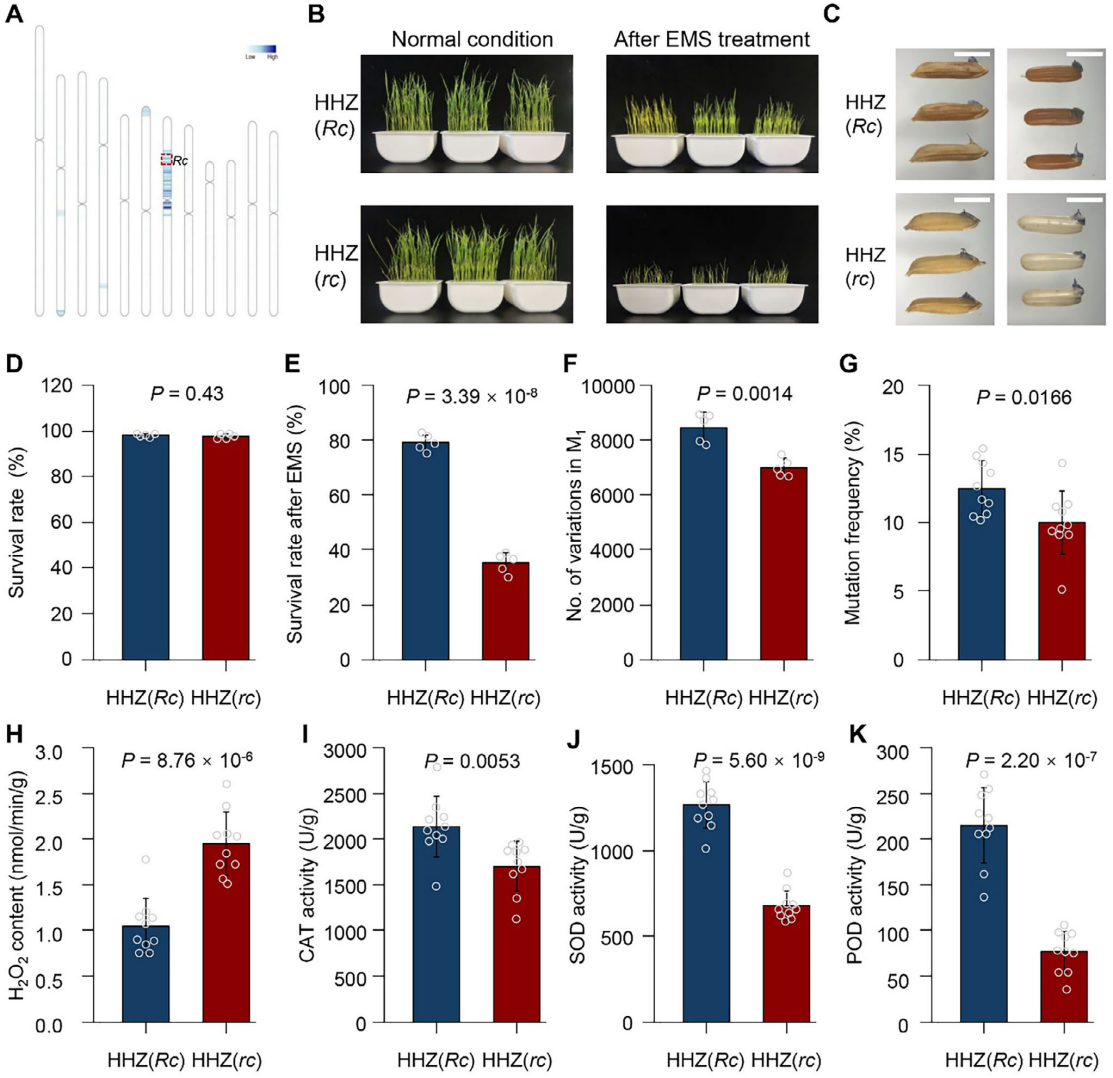
The *Rc* gene enhances EMS mutagenesis efficiency by improving antioxidant capacity in rice seeds. A) Genetic background analysis of *Rc* near‐isogenic lines (NILs) in the HHZ background. B) Representative seedling survival images derived from seeds before and after EMS mutagenesis. C) Brown rice appearance following NBT staining of EMS‐treated seeds. D,E) Comparison of seedling survival rates under normal conditions (D) and after EMS mutagenesis (E). F,G) Comparison of the number of EMS‐induced SNPs in M_1_ plants (F) and mutant frequency in M_2_ plants (G). H–K) Comparison of H_2_O_2_ content (H), and enzyme activities of CAT (I), SOD (J), and POD (K) in seeds after EMS mutagenesis. All *P*‐values for significance were calculated using two‐tailed Student's *t*‐tests.

To further verify this finding in a uniform genetic background, we developed near‐isogenic lines (NILs) in the Huanghuazhan (HHZ) background, designated as HHZ (*rc*) and HHZ (*Rc*). Apart from the *Rc* locus, the two lines share nearly identical genetic backgrounds and exhibit no significant differences in major agronomic traits, except for pericarp color (**Figure** **5**A; Figure S5, Supporting Information). Under normal growth conditions, seed survival rates were not significantly different between HHZ (*rc*) and HHZ (*Rc*) (Figure 5C,D). In contrast, after EMS treatment, HHZ (*Rc*) had a significantly higher seed survival rate than HHZ (*rc*) (Figure 5E), indicating that *Rc* significantly enhances seed tolerance to EMS.

Haplotype analysis further showed that over 91% of the Aus subpopulation carries functional *Rc* alleles, consistent with not only the superior EMS tolerance but also the elevated mutagenesis frequency observed in *Aus* rice (Figures 2, 4, 3A–C). To test whether *Rc* directly affects mutagenesis frequency, we performed whole‐genome sequencing of M_1_ plants from HHZ (*rc*) and HHZ (*Rc*). HHZ (*Rc*) exhibited significantly higher numbers of EMS‐induced mutations than HHZ (*rc*) across the genome (Figure 5F). Consistently, in the M_2_ generation, the frequency of visible mutants was also significantly higher in HHZ (*Rc*) than in HHZ (*rc*) (Figure 5G). These findings demonstrate that functional *Rc* alleles not only improve EMS tolerance but also increase mutagenesis efficiency in rice.

To explore the underlying mechanism for *Rc* to enhance EMS tolerance, we first examined the accumulation of superoxide in embryos after EMS treatment using NBT staining. The embryos of *Rc* seeds showed lighter staining than those of *rc* seeds, indicating higher embryo cell viability in *Rc* seeds (Figure 5C; Figure S6A, Supporting Information). Measurement of reactive oxygen species (ROS) levels confirmed that *rc* seeds accumulated significantly higher levels of H_2_O_2_ and other ROS than *Rc* seeds (Figure 5H; Figure S6B, Supporting Information). Since antioxidant enzymes play a crucial role in ROS detoxification, we assessed the activities of catalase (CAT), superoxide dismutase (SOD), and peroxidase (POD). All enzymes exhibited significantly higher activities in *Rc* seeds than in *rc* seeds (Figure 5I–K; Figure S6C–E, Supporting Information). Consistently, both germination potential and final germination rate were significantly higher in *Rc* than in *rc* genotypes after EMS treatment (Figure S7, Supporting Information), indicating that *Rc* has a higher seed cell viability than *rc*. Given that *Rc* is known as a major regulator of proanthocyanidin biosynthesis in the rice pericarp, we propose that *Rc* enhances EMS tolerance and mutagenesis efficiency by elevating antioxidative capacity, thereby mitigating EMS‐induced oxidative damage.

## 3 Discussion

Chemical mutagenesis with EMS is a powerful tool for generating genetic diversity and dissecting gene function in crops. Globally, large numbers of mutagenized resources have been developed to support breeding research. According to the FAO/IAEA mutant variety database (https://mvd.iaea.org), more than 1,350 mutagenized rice varieties have been officially released. Compared with physical mutagens, EMS is particularly effective for generating high‐density point mutations, so that has been successfully used to create large‐scale mutant libraries. For example, ≈60 000 mutant lines have been generated in the indica rice variety IR64 through combined EMS and irradiation, which have greatly accelerated functional genomic studies.^[^ ^13^ ^]^ To maximize efficiency, EMS treatments are generally calibrated to induce 50–80% lethality^[^ ^5^, ^20^ ^]^ for ensuring adequate mutation accumulation while maintaining an acceptably viable seed population. However, most studies implicitly assume that rice varieties respond uniformly to mutagenesis under a given treatment regime. In contrast, our systematic comparison of 420 genetically diverse accessions revealed striking EMS‐induced variation in both plant tolerance and mutation frequency, where *Aus* rice was the best among subpopulations. These findings highlight the necessity of tailoring EMS protocols to the genetic background of target materials, rather than relying on uniform treatment conditions.

The enhanced EMS tolerance conferred by the functional *Rc* gene likely reflects its role in boosting seed antioxidant capacity. Functional *Rc* promotes the accumulation of flavonoid compounds, such as proanthocyanidins, in the seed coat, and these flavonoids possess strong free radical‐scavenging activity (Prasad et al., 2023; Rebeira et al., 2024). During EMS mutagenesis, which elevates reactive oxygen species (ROS), *Rc* seeds had higher SOD and POD activity and accumulated significantly less H_2_O_2_ than *rc* seeds, thereby mitigating oxidative damage to DNA and cellular structures to result in the seeds with higher viability. This aligns with the reports that the proanthocyanidins in functional *Rc* seed coats enhance seed survival under oxidative stress.^[^ ^21^ ^]^ While our data strongly support an antioxidant‐mediated mechanism, we cannot exclude alternative or complementary roles, such as potentially indirect effects of *Rc* on DNA repair pathways. *Rc* is known to regulate flavonoid and ABA biosynthesis,^[^ ^22^ ^]^ but whether it also affects genomic repair systems remains an open question. Interestingly, prior studies have shown that exogenous application of anthocyanins or crop materials with high anthocyanin accumulation capacity can improve seed survival under high‐energy radiation, a stress condition analogous to the oxidative stress induced by EMS in this study. Therefore, the most parsimonious explanation is that the *Rc* enhances the tolerance to EMS or reduces oxidative damage primarily by elevating antioxidant capacity via flavonoid metabolism.

Interestingly, a positive correlation was observed between antioxidant capacity and EMS‐induced mutation load (Figure 5D–G), which might appear counterintuitive because reactive oxygen species (ROS) are typically associated with DNA damage. A plausible explanation for this phenomenon involves differences in embryonic cell survival following mutagen exposure. We propose that the elevated oxidative stress in HHZ(*rc*) seeds leads to extensive cytotoxicity, eliminating a large fraction of mutagenized M_0_ embryonic cells, and thereby narrowing the genetic bottleneck through which mutations are transmitted to the M_1_ generation. In contrast, the enhanced antioxidant system in HHZ(*Rc*) mitigates oxidative damage, enabling a broader survival of M_0_ cells. As a result, a more representative spectrum of primary EMS‐induced mutations—originating mainly from direct guanine alkylation rather than oxidative damage—is preserved and subsequently detected in M_1_ plants. By analyzing the EMS‐induced SNP spectra in HHZ(*Rc*) and HHZ(*rc*), we found no significant differences in the G/C‐to‐A/T mutation ratios or the distribution of other mutation types between the two genotypes (Figure S7C, Supporting Information). This supports our hypothesis and further indicates that the higher mutation count observed in HHZ(*Rc*) is more likely attributable to enhanced mutation retention resulting from improved cell viability, rather than an intrinsically higher initial mutation rate.

Our results also reveal that rice varieties carrying functional *Rc* alleles exhibit higher mutagenesis efficiency, which can be practically utilized to optimize mutagenesis breeding. Parents with high antioxidant capacity, such as those with functional *Rc*, could be prioritized for EMS mutagenesis to generate rich mutant allele pools. Our study demonstrated that over 91% of the Aus accessions carry functional *Rc* haplotypes, consistent with their strong EMS tolerance and elevated mutation frequency. Molecular markers, such as those detecting the H1 haplotype in exon 6 of *Rc*, could streamline parental selection. As noted by Rebeira et al.,^[^ ^23^ ^]^ such markers reliably identify red‐grained lines with high antioxidant activity, offering an efficient alternative to labor‐intensive biochemical assays. However, breeding applications must account for the pleiotropic effects of *Rc*. Red pericarp is strongly linked to seed dormancy, which may be undesirable in developing high‐yielding white‐grained cultivars where rapid and uniform germination is essential. *Rc* also contributes to pigmentation and pre‐harvest sprouting resistance,^[^ ^22^ ^]^ further complicating its use in conventional breeding. To balance these trade‐offs, biotechnological strategies may be preferable, for instance, introducing anthocyanin biosynthesis genes into elite white rice backgrounds to mimic *Rc*‐like antioxidant capacity, or applying exogenous antioxidant treatments to seeds before or after mutagenesis. In conclusion, when functional *Rc* is used to improve mutagenesis efficiency, breeders must make a good balance of its pleiotropic effects for maximizing its value, contributing to breeding objectives.

Finally, our study on rice adds to a growing body of evidence from Arabidopsis and barley showing that EMS responses are highly genotype‐dependent, where different varieties display contrasting mutagen tolerance and mutation frequency under identical treatment conditions.^[^ ^8^, ^15^ ^]^ By performing a relatively large‐scale comparative analysis in a mutation study, we not only document substantial variation across subpopulations of cultivated rice but also characterize new roles of *Rc* through GWAS for improving efficiency in cultivar development. Collectively, our findings underscore the importance of genetic background in shaping EMS mutagenesis outcomes and provide theoretical and practical support for precision‐oriented mutagenesis breeding strategies in rice.

## 4 Experimental Section

### Plant Materials

The *indica* rice variety Yuehuangjinzhan (YHJZ) and *japonica* variety K9553 were initially cultivated in 2020 at the Huimin Experimental Base of Sichuan Agricultural University (Chengdu, Sichuan) as the parental generation (P_0_). Seeds harvested from these plants (P_1_ generation) were subjected to EMS mutagenesis at concentrations of 0.6%, 1.2%, and 1.8% for 16, 20, and 24 h, respectively. The resulting P_1_M_1_ seeds were planted in the winter of 2022 at the Southern Breeding Base of Sichuan Agricultural University (Lingshui, Hainan) to establish a mutant population. P_2_M_2_ seeds, harvested from individual P_1_M_1_ plants, were sown in family‐based arrangements in 50‐cell trays in the summer of 2023 to quantify mutant frequency under each treatment condition.

A diverse panel of 420 cultivated rice accessions was used for population analysis, encompassing 38 Aus, 143 IndI, 119 IndII, 60 TeJ, and 46 TrJ varieties previously described in earlier studies.^[^ ^24^, ^25^ ^]^ All materials were grown in the winter of 2022 at the Southern Breeding Base (Lingshui, Hainan). Harvested seeds (P_1_ generation) were treated with 0.6% EMS for 20 h in the winter of 2023. The resulting P_1_M_1_ seeds were planted at the same base to assess seedling survival rates before and after mutagenesis. Mature plants (P_2_M_2_ generation) were individually harvested, their seeds were sown in family‐based 50‐cell trays in the summer of 2024 to determine mutant frequency.

Near‐isogenic lines (NILs) of HHZ (Rc) and HHZ (rc) were developed by crossing Huanghuazhan (HHZ) with the red‐pericarp variety Hongainuo, followed by six successive backcrosses with HHZ to generate BC_6_F_1_ progeny. From the segregating BC_6_F_2_ population, individuals homozygous for either the functional *Rc* or non‐functional *rc* allele but displaying no significant differences in major agronomic traits were selected as NILs. Genotyping during NIL development was performed using a molecular marker targeting the 14‐bp functional indel in *Rc* (Table S4, Supporting Information).^[^ ^18^ ^]^ The selected NILs were validated by whole‐genome resequencing to confirm genetic background purity. Both HHZ (*Rc*) and HHZ (*rc*) were treated with 0.6% EMS for 20 h to generate M1 seeds. The M1 plants were cultivated in the summer of 2025 at the Huimin Experimental Base (Chengdu, Sichuan) to assess seedling survival rates, and their M_2_ seeds were sown individually in 100‐cell trays to quantify mutant frequency.

Knockout mutants of *Rc* were generated using the CRISPR/Cas9 system as previously described.^[^ ^26^ ^]^ Two target sites for *Rc* were designed using the TargetDesign web tool, and the corresponding primers (Rc‐Target1‐U6a and Rc‐Target2‐U6b, Table S4, Supporting Information) were used to construct sgRNA expression cassettes driven by U6a and U6b promoters. These cassettes were assembled by overlapping PCR using pYLsgRNA‐OsU6a and pYLsgRNA‐OsU6b as templates, and inserted into pYLCRISPR/Cas9P_ubi_‐H vector to generate the final *Rc*‐KO construct.

### EMS Mutagenesis and Phenotypic Identification

EMS mutagenesis was performed using the mutagen ethyl methanesulfonate from SIGMA (Product No. M0880). To determine optimal mutagenesis conditions, combinations of three EMS concentrations (0.6%, 1.2%, and 1.8%) and three treatment durations (16, 20, and 24 h) were tested, yielding nine treatments with three replicates each. Quantified batches of YHJZ and K9553 seeds were placed in mesh bags, pre‐soaked in distilled water for 24 h at room temperature, and then treated with EMS solutions at the specified concentrations. After treatment, the seeds were thoroughly rinsed under running water for 2–3 h and sown, together with untreated controls, in labeled soil plots. Seedling emergence at the three‐leaf stage was recorded to determine optimal mutagenesis conditions.

For the 420 diverse accessions, 600 well‐filled seeds from each were equally divided into six portions, each with 100 seeds. Three served as untreated controls, and the other three were treated with 0.6% EMS for 20 h. After treatment, the mutagenized and control seeds were sown similarly in demarcated soil plots, and seedling emergence was recorded at the three‐leaf stage. Then, the seedlings were transplanted and grown to maturity, and a single panicle was harvested from each plant.

The frequency of visible leaf‐color mutants was commonly used as a proxy for estimating mutation rates.^[^ ^27^ ^]^ For each accession, 100 M_1_ plants (or all available plants if fewer) were individually harvested, and ≈50 M_2_ seeds from each M_1_ plant were sown in tray cells. At the M_2_ seedling stage, each cell was recorded for albino, lesion mimics, or withered leaf tips. The proportion of mutant‐containing cells relative to the total number sown was used to estimate mutant frequency.

### Genome‐Wide Identification of EMS‐Induced SNPs

Whole‐genome sequencing was used to identify SNPs induced by EMS mutagenesis in M_1_ mutant individuals. To compare the distribution of EMS‐induced SNPs among rice subpopulations, M_1_ plants were randomly sampled from five subpopulations—Aus, IndI, IndII, TeJ, and TrJ—comprising 5, 11, 9, 5, and 5 accessions, respectively. For each accession, leaves from five M_1_ plants were pooled in equal amounts for sequencing. To assess the effect of *Rc* genotype on EMS‐induced SNP accumulation, leaf samples from five M_1_ plants each of HHZ (*Rc*) and HHZ (*rc*) were sequenced. All M_1_ mutant individuals and wild‐type individuals were resequenced using the Illumina HiSeq platform (PE150/250, insert size 300–500 bp).

Identification of EMS‐induced SNPs followed the pipeline of Yan et al.^[^ ^28^ ^]^ After quality filtering, resequencing data from each mutant pool (average coverage ≈15×) were aligned to the Nipponbare reference genome (MSU v7.0) to identify all SNPs in each pool.^[^ ^29^ ^]^ The detected SNPs included both standing natural variations and EMS‐induced mutations. To minimize false positives arising from sequencing errors, only SNPs meeting the following criteria were retained as reliable EMS‐induced mutations: 1) Absent from known natural variant sites reported in the 3K‐RGP database;^[^ ^30^ ^]^ 2) Supported by ≥3 reads with base quality ≥20 in the mutant sample, and by ≤1 supporting read across all other mutant samples; and 3) Unique to only one mutant sample and absent from all others.

### Functional Annotation of Genetic Variants

To functionally prioritize the identified genetic variants, variant annotation and classification were performed using snpEff software.^[^ ^31^ ^]^ Each variant was categorized into one of four impact levels based on its predicted effect on gene function: HIGH impact ‐ Variants predicted to severely disrupt gene function, typically causing major alterations in the gene product. This category includes start‐gain, start‐loss, stop‐gain, stop‐loss, and splice‐site variants, all of which were likely to abolish or substantially impair protein function. MODERATE impact ‐ Variants expected to alter protein effectiveness without complete loss of function, such as non‐synonymous (missense) mutations that substitute one amino acid for another, potentially affecting protein stability, structure, or interactions. LOW impact: Variants predicted to have minimal influence on protein function, primarily synonymous substitutions that do not change the encoded amino acid sequence. MODIFIER impact: Variants located in non‐coding or regulatory regions, such as intergenic, intronic (except at splice sites), and untranslated regions, which may affect gene expression or transcript regulation, but not directly alter protein structure. The genomic distribution of each SNP category was visualized using the ‘circlize’ package in R.^[^ ^32^ ^]^

### Genome‐Wide Association Study and Gene Haplotype Analysis

A genome‐wide association study (GWAS) was performed for seven phenotypic traits: Albino seedling rate (ASR), Lesion mimic rate (LMR), Leaf tip necrosis rate (LTNR), All mutant type (AMT), Normal survival rate (NSR), Survival rate after EMS (SRAE), and Survival rate decrease (SRD). These traits were grouped into two categories: EMS tolerance‐related traits (NSR, SRAE, SRD) and mutation frequency‐related traits (ASR, LMR, LTNR, AMT). Raw phenotypic data (Data S1, Supporting Information) were subjected to a BOX‐COX transformation prior to GWAS analysis to ensure normal distribution.

The 420 rice accessions used for GWAS were previously sequenced and genotyped.^[^ ^33^ ^]^ GWAS for EMS mutagenesis efficiency traits was performed using a mixed linear model (MLM) that incorporated both population structure and relative kinship as covariates. Only variants including SNPs and InDels with a minor allele frequency (MAF) > 5% and a missing rate < 15% were retained, resulting in 6.3 million variants (5.5 million SNPs and 0.8 million InDels) for downstream GWAS analyses.

The genome‐wide significance threshold was determined using a modified Bonferroni correction,^[^ ^34^ ^]^ where the total SNP number (M) was replaced with the effective number of independent SNPs (Me). The MLM‐based analysis was implemented using EMMAX software.^[^ ^35^ ^]^ Variant positions were based on the Nipponbare reference genome (MSU v7.0),^[^ ^29^ ^]^ with data obtained from http://rice.uga.edu/.

For linkage disequilibrium (LD) visualization, SNPs and InDels (MAF > 0.03) located within a 400 kb region upstream and downstream of each peak SNP were used to generate LD heatmaps with the R package “LDheatmap”.^[^ ^36^ ^]^ For haplotype analysis of the *Rc* gene, genotype data of variants located within its coding region were downloaded from the RiceVarMap 2.0 database (http://ricevarmap.ncpgr.cn).^[^ ^37^ ^]^ The variants with MAF < 0.05 or a missing rate > 10% were excluded to ensure haplotype accuracy. Only haplotypes represented by at least five accessions were retained for visualization of haplotype diversity and frequency distribution.

### Reactive Oxygen Species and Antioxidant Enzyme Assays

ROS accumulation in germinated rice seeds was assessed using nitroblue tetrazolium (NBT) staining. Seeds were vacuum‐infiltrated for 20 min and stained with NBT solution in the dark until both ends of the seeds turned blue. The stained seeds were fixed in Carnoy's solution, followed by decolorization in a 90% ethanol water bath until the tissues became colorless, and then stored in glycerol solution. Samples were observed and imaged using a stereomicroscope. Antioxidant enzymes, including superoxide dismutase (SOD), catalase (CAT), and peroxidase (POD), were quantified following the methods of Zhou et al.^[^ ^17^ ^]^

### Statistical Analysis

All statistical analyses were conducted in R.^[^ ^38^ ^]^ Histograms, boxplots, correlation analyses, and multiple comparisons were performed using the mean phenotypic values from three biological replicates per accession. Regression between mutant frequency and survival rate was analyzed using the ‘lm’ function in R. Two‐tailed *t*‐tests were used to compare mutagenesis efficiency traits between genotypes. Pearson correlation coefficients were calculated using the cor.test() function, with *P*‐values determined by two‐tailed *t*‐tests. Multiple comparisons of phenotypic data among subpopulations or haplotypes were performed with Duncan's multiple range test via the duncan.test() function from the “agricolae” R package,^[^ ^39^ ^]^ using a significance threshold of *P*<0.01. Boxplots were generated using the base boxplot() function in R to visualize significant group differences.

## Conflict of Interest

The authors declare no conflict of interest.

## Author Contributions

P.X., Q.Z., and L.X. contributed equally to this work. P.X., Q.Z., L.X., D.X., S.Z., Z.Z., N.L., and Z.C. performed the experiments. P.X. and H.Z. conceived and designed the experiment. X.W., and H.Z. wrote and edited the article.

## Supporting information



Supporting Information

Supporting Table

## Data Availability

The data that support the findings of this study are available in the supplementary material of this article.
