# Supplementary material for: Genome‐Wide Association Analyses Reveal the Genetic Basis of EMS Mutagenesis Efficiency in Rice
Source: Adv Sci (Weinh). 2025 Nov 20;13(8):e17647. doi: 10.1002/advs.202517647 (PMC12884769; doi:10.1002/advs.202517647)
Supplement: Supplementary file 1 — Supporting Information [file ADVS-13-e17647-s001.docx]

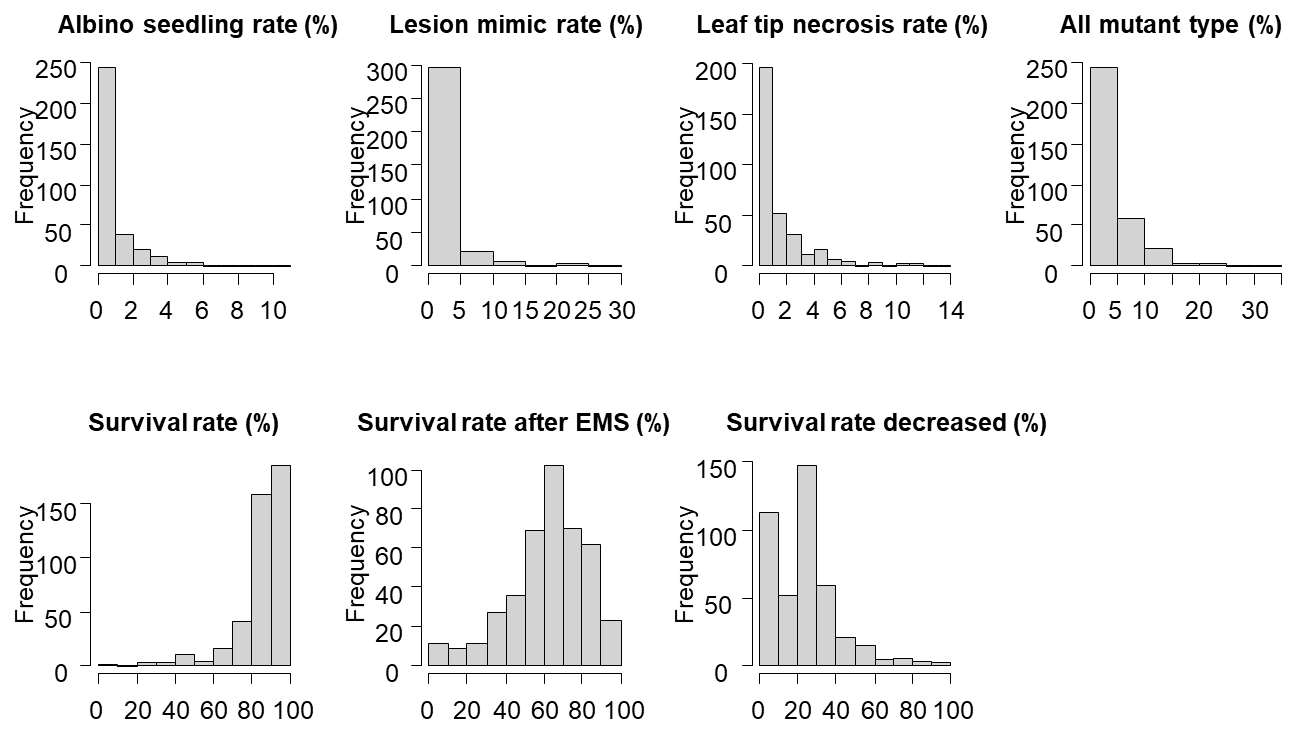


**Figure S1.** Frequency distributions of mutation efficiency traits among 420 cultivated rice accessions. The X-axis represents the trait value range, and the Y-axis shows the frequency of accessions within each interval. Detailed definitions of each trait are provided in the Experimental Section.


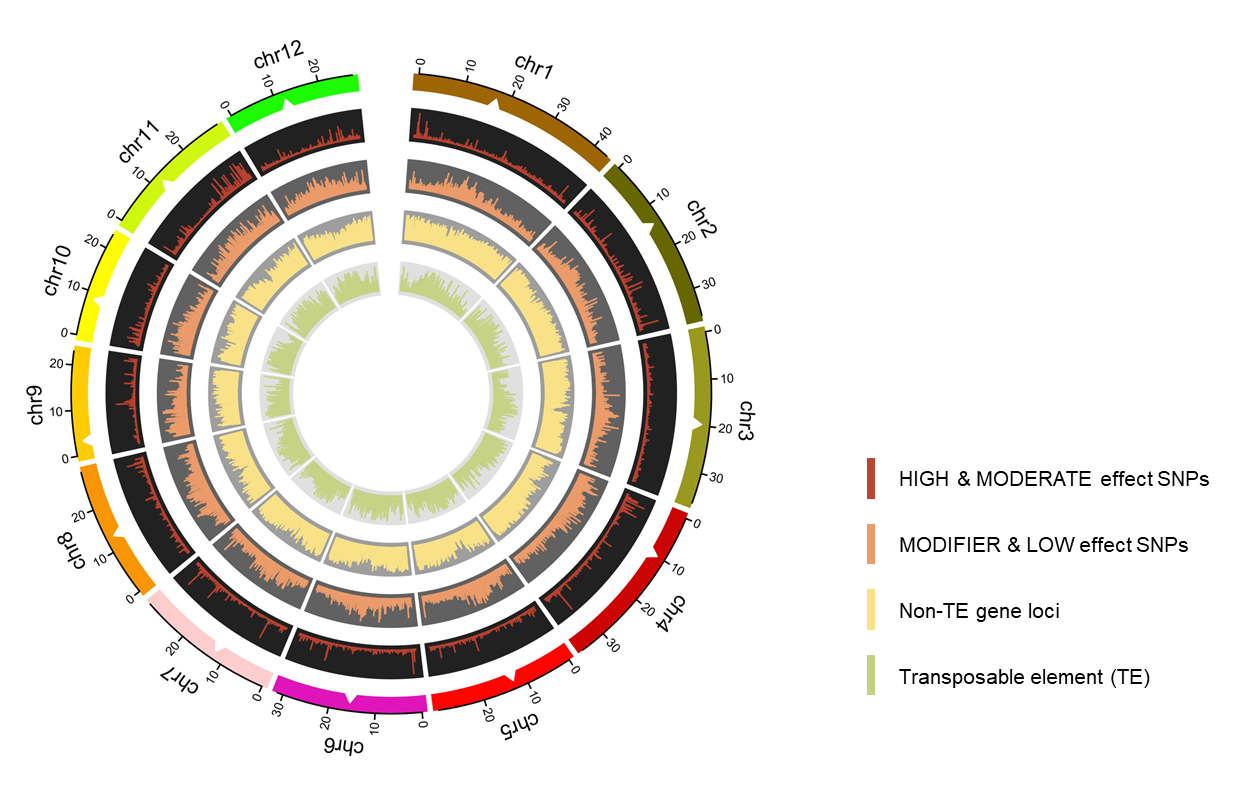


**Figure S2.** Genome-wide distribution of EMS-induced SNPs and gene loci in rice. From the outside inward, the outermost circle represents the chromosomes, followed by HIGH & MODERATE effect SNPs, MODIFIER & LOW effect SNPs, Non-TE gene loci and TE elements. The line height in each circle indicates the frequency of SNP variation or genes across chromosomal regions for each subpopulation. Definitions of the four SNP effect categories of ‘HIGH’, ‘MODRATE’, ‘LOW’ and ‘MODIFIER’ are provided in the Experimental Section.


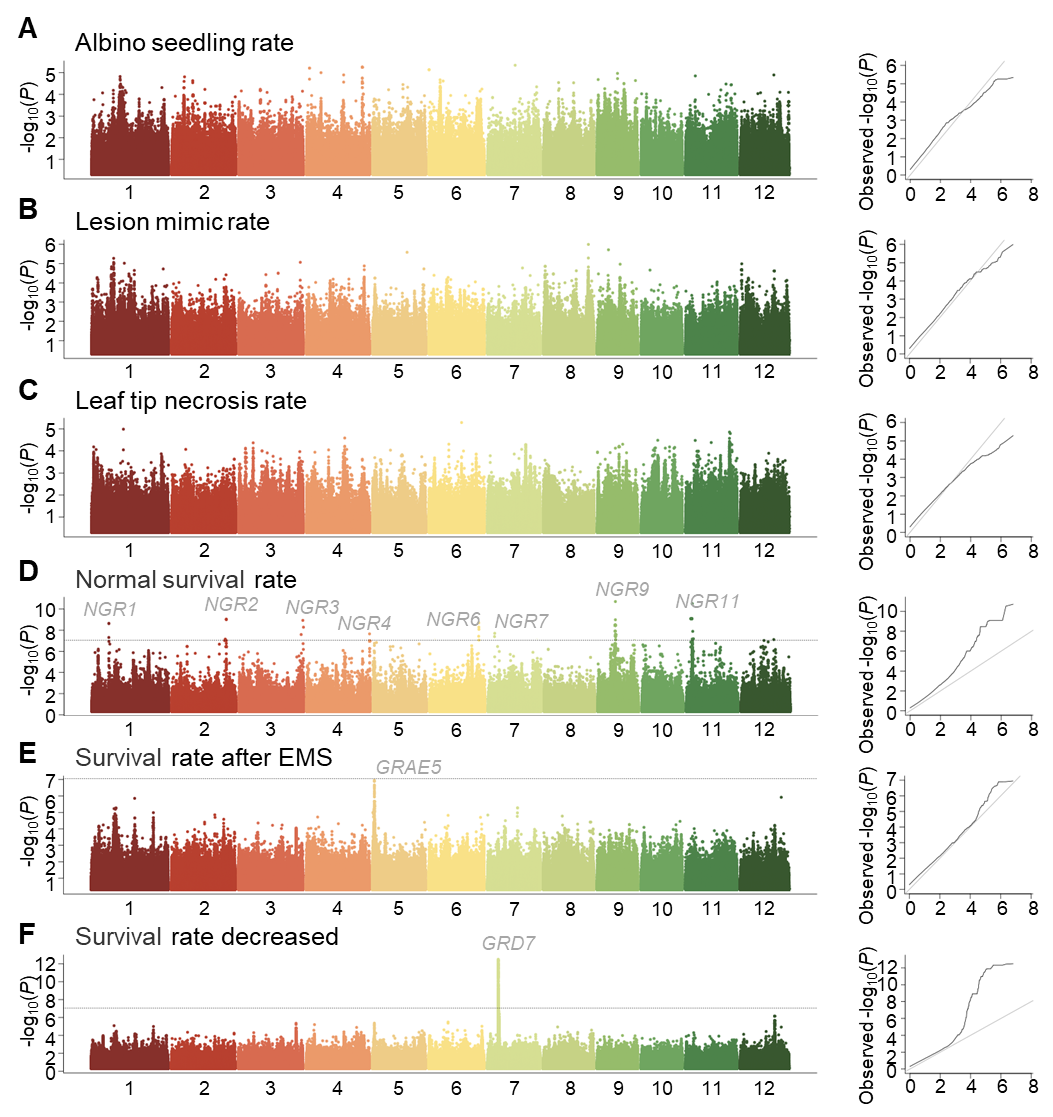


**Figure S3.** Manhattan and quantile–quantile (Q-Q) plots of GWAS for seven EMS mutagenesis efficiency traits. The X-axis denotes the physical positions of SNPs across the 12 rice chromosomes, and the Y-axis shows the -log_10_(*P*) values.


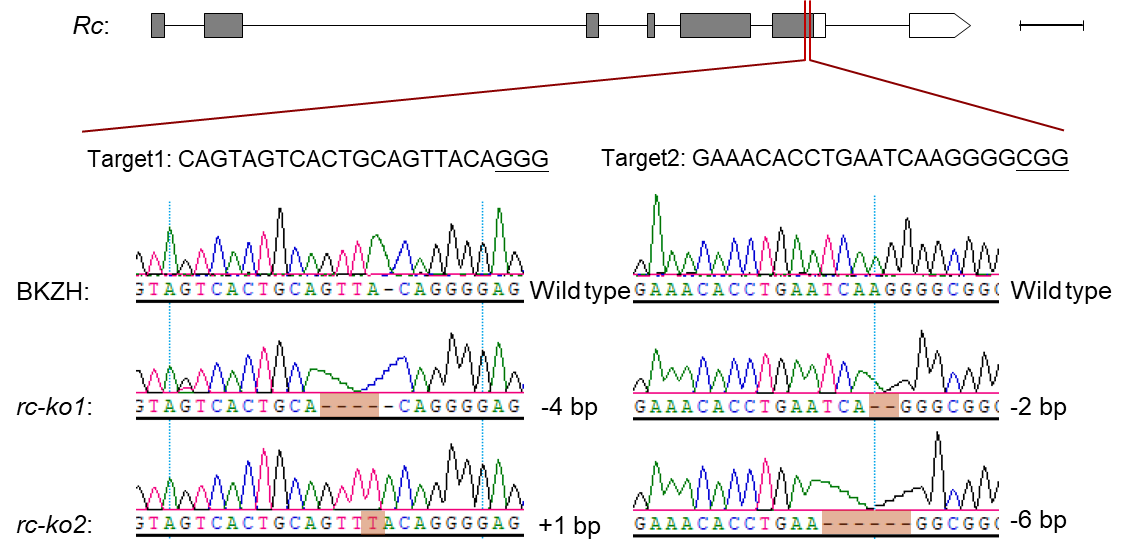


**Figure S4.** Gene structure of *Rc* and CRISPR/Cas9 target sites. Red lines indicate the position of guide RNA (scale bar = 500 bp). Sequence alignments of BKZH and knockout mutants (*rc-ko1* and *rc-ko2*) for *Rc* show the induced mutations highlighted in pink boxes.


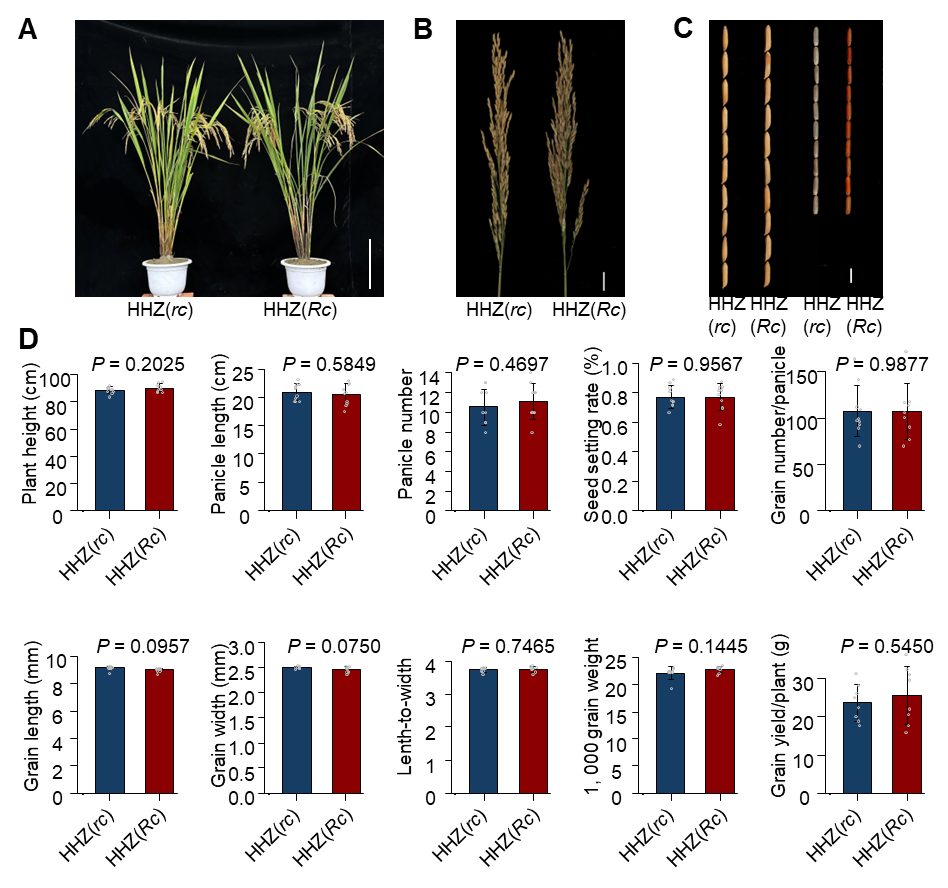


**Figure S5.** Comparison of agronomic traits in near-isogenic lines for *Rc* gene. (A-C) Comparison of plant type (A), panicle length (B), and seed and brown rice appearance (C) between HHZ(*rc*) and HHZ(*Rc*). (D) Comparison of agronomic traits. All *P*-values for significance were calculated using two-tailed Student’s *t*-tests.


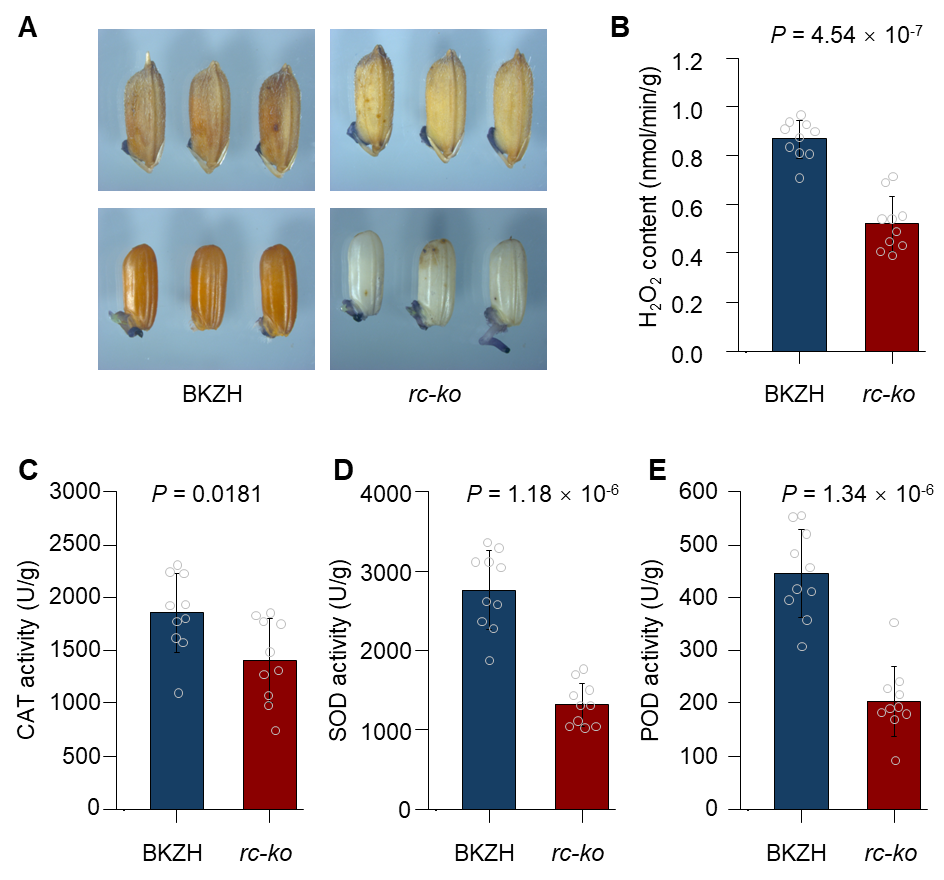


**Figure S6.** Knockout of *Rc* gene reduces antioxidant capacity in rice seeds. (A) Comparison of seed and brown rice phenotypes in BKZH and knockout lines after NBT staining following EMS mutagenesis. (B-E) Comparison of H₂O₂ content (B), CAT activity (C), SOD activity (D), and POD activity (E) in seeds post-EMS treatment. All *P*-values for significance are based on two-tailed *t*-tests.


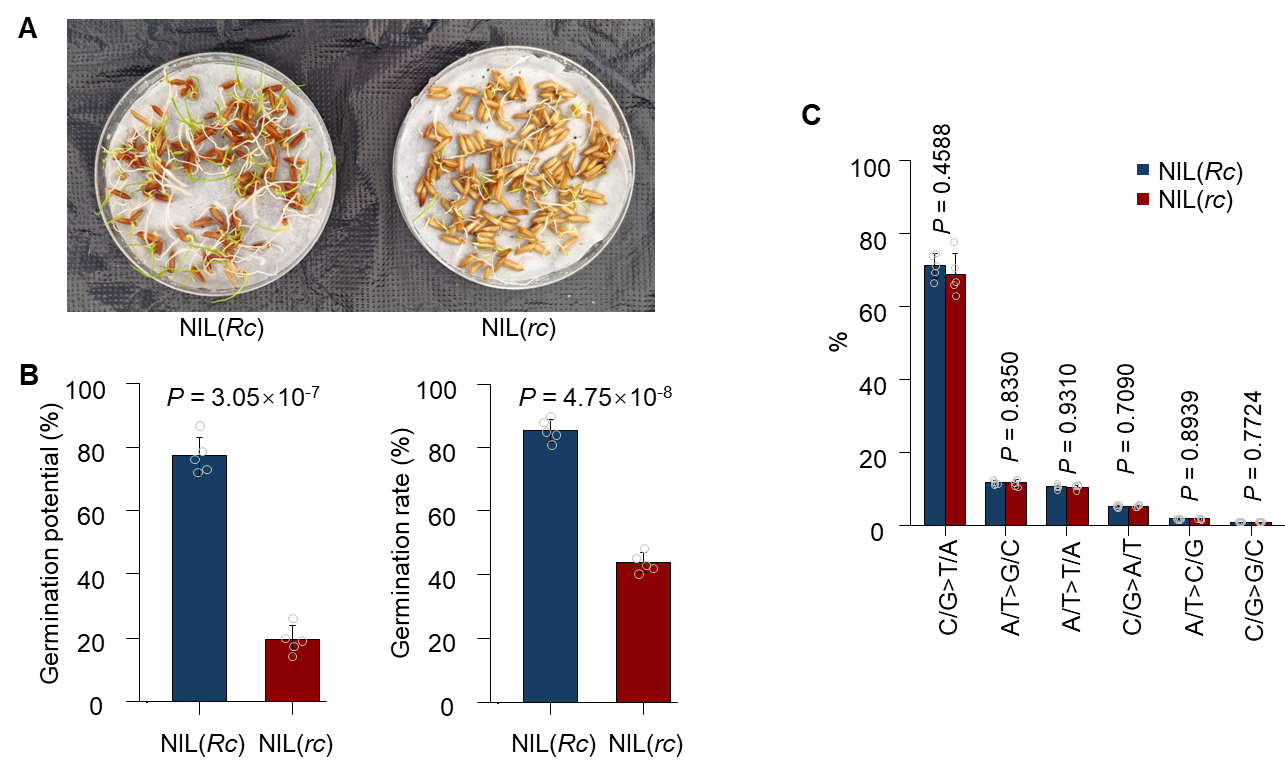


**Figure S7.** Effects of *Rc* genotype on seed germination and EMS-induced mutation spectra. (A) Representative image showing germination performance after EMS treatment. (B) Comparison of germination potential (left) and final germination rate (right) between near-isogenic lines (NILs). (C) Spectrum of EMS-induced single-nucleotide transitions in *Rc* and *rc* NILs. All *P*-values for significance are based on two-tailed *t*-tests.
